# Practical Hints on the Treatment of Several Diseases

**Published:** 1834-10-01

**Authors:** 


*Practical Hints on the Treatment of several Diseases.* By John Peacock, m.d.—London, 1834. 8vo. pp. 77.

This is a collection of strange cases, queerly narrated by a doctor who is evidently rather an odd fish, but nevertheless a sound and sensible practitioner. Dr. Peacock writes in a slashing, old-fashioned style, as if he had sailed in the capacity of surgeon with Commodore Trunnion or Lieutenant Hatchway; but his cases are told with so much candour, and his practice is so much to the point, that, if we ever ventured so far north, and found ourselves seized with some critical distemper in the County Palatine, we should have no hesitation in entrusting the care of our reviewing selves to Dr. Peacock.

The work commences with several cases of mortification, treated with opium, ammonia, and aromatics: of course, we have nothing to say against this plan, but we are surprised that our author considers it as original. In giving the first case, cured in this manner, he says, "I here merely put out my FEELERS, having had some peculiar opinions concerning mortification some time, resolving to take the first opportunity of putting them to the test." (P. 9.) The following is another of these cures.

"An old sailor who had served his Majesty a great number of years, in every variety of climate, came to the Dinsdale sulphur baths to seek relief from a violent pain in both legs, but more particularly in one of them. As I attended frequently at this delightful spring, I was sometimes applied to by this poor man, and I administered to relieve his sufferings, but often in vain: as the cold weather advanced his sufferings increased, and grown desperate, he cut down upon the metatarsal bones of his worst foot with a penknife, with the view, as he said, to bottom the complaint. A mortification immediately ensued, and as he had been frequently assisted by some kindhearted ladies at the hotel, chiefly of Lord Dundas' family, as I was not there the first day, they were good enough to procure him two surgeons. As soon as I heard of it I called upon the man, and found his leg was condemned, as the surgeons had told him he must lose his limb or his life. I am sorry that I could not attend more to professional etiquette. I was vexed at the man being taken from me, and being conscious this was a case I was perfectly master of, I was provoked to tell him that if he remained with me I could cure him. I have related this gossip, just to shew that it was considered a very serious case. There was nothing remarkable in the recovery. The bones of the great toe were so completely denuded of their periosteum, that they were obliged to be removed, and the restoration of the limb was rather slower than I expected; but it is not to be wondered at, when a constitution is based upon grog, which I dare say is frequently the case among the sailors in the navy. The medicine used on this occasion was from the formulæ in the first page, with now and then a slight alteration.

"The man walked well when he set off for his home, at South Shields. I should not omit that the family of Lord Dundas stood the whole expense." (P. 15.)

Here is a case of diabetes successfully treated.

"I was consulted by a military gentleman, about fifty, who was a good deal wasted by the complaint: he had a craving appetite, much thirst, and a dull pain in the region of his stomach, which he could not call heartburn, but it was nearly allied to it, for he had formerly suffered a great deal from the heartburn; his skin was dry, and his urine, he said, had long tasted sweet; it was pale, and measured about six quarts in the twenty-four hours, sometimes more; the pulse was feeble, but not very quick; the tongue red, and very dry—such a tongue as is frequently seen in the typhus fever; he had a trifling cough, such as is generally called *symptomatic;* his bowels were very confined. We began with ten leeches applied to the stomach, and a couple of the common aperient pills, to be repeated every two hours till a passage is procured. The next day the following powder was ordered, to be repeated every four hours, night and day, in spring water:—℞. Ferri præcip. gr. v.: Opii, gr. ss. ad gr. i.; Pulv. Aromat. gr. iij.; Cretæ pp. ʒi.; Pulv. Gum Arabic. ʒi.; Pulv. Jacobi, gr. iij. M. ft. pulvis.

"The half-grain of opium in this prescription was not inserted as an astringent, but, from my experience, nothing prevents the acid fermentation of the stomach so certainly as a small dose of opium, or Extract. Papav. Alb. This gentleman lived some distance from me, but he left me with directions that he was to please his appetite in what he eat, making a point that animal food should prevail when he could make it agreeable to his taste, and he was SWORN to take only water to drink.

"A fortnight was given him to try the effects of the medicine, and then to let me see him; but he wrote to me in the course of a week, begging me not to forget the prescription; he said I should see a gay young fellow at the end of the fortnight. When he came, there was indeed a strange alteration; from a withered old fellow he had become corky and facetious, and begged me to fix the day when we could have a bottle of wine together. I ordered him to be bled with leeches the next day, and then again a fortnight after that; and from this time he rapidly got well, without any alteration in his medicine, except increasing the opium a little, but never to exceed a grain, and I had to vary the aperient two or three times to please his whims. I think, from first to last, he took the cretaceous medicine about six weeks; but he confessed that he sometimes, towards the latter end, lengthened the interval between the doses.

"I have got to place implicit reliance upon this plan of medicine, when we have our patients tolerably early; but they too frequently apply when the tone of the stomach and other viscera are entirely destroyed, and a hopeless emaciation has taken place." (P. 20.)

Dr. Peacock gives a second case, in which the disease alternated with anasarca, and the patient died under the care of another practitioner; and a third, in which the patient was cured.

There is probably no reader of these pages who is ignorant of the fact so ably set forth by Gooch, Marshall Hall, and others, that pain from exhaustion may simulate inflammatory pain so accurately as to deceive even skilful practitioners; but as this truth, though known, is often neglected, we offer no apology for the insertion of the following cases.

"*Pain from Exhaustion.* Whether I designate this chapter aright I cannot say, as I am so little disposed to theorise; and those who like this employment may settle it their own way: it is from the mode of cure that I have taken up the idea.

"A gentleman, apparently between forty and fifty, came to our popular watering-place, Middleton, with a violent deep-seated pain in his head; which, upon any exertion, as walking, or lifting even a moderate weight, threw him into convulsions, apparently of the epileptic kind. I found, upon enquiry, he had had some troubles upon him, which he had dwelt upon a good deal. His pulse was about eighty-six, and neither hard nor strong; the eyes were not red, but the pupils were a little larger than we generally meet with in health; the urine sometimes very pale, and in good quantity; from what I could learn, the depletory system had been carried to some extent; after emptying his bowels, I gave him the following powder, to be repeated every six hours.—℞. Pulv. Ipecac. Comp.

gr. vj.; Pulv. Aromat. gr. iij.; Calomel, Opii. Pulv. ana. gr. ss. M. ft. pulv.

"This medicine released him from his sufferings very soon, so that in a few days he was enabled to go home.

"I was called in to consult upon the case of a carpenter, in Piersebridge, who had long suffered under an intense pain in the head, which had, with some reason, been supposed to proceed from occasional inflammation of the membranes of the brain. The pupils of his eyes were rather contracted; his pulse from a hundred to a hundred and ten, when the pain was bad; and, when it was upon him, a sudden shake, or a false step, was very inconvenient. The depletory practice was pushed to no purpose. This case was a puzzle; but the compound ipecacuanha, with small doses of opium and calomel, restored him.

"A near neighbour to me had a fine well-grown child, about three years old, who had been observed to droop a good deal during a fortnight or more; a strong light, or a noise, both offended him; he frequently was observed to rest his head upon one of the chairs, and often screamed suddenly, putting his hand to his head; his bowels were costive, and his stools very dark; his pulse ran two or three changes during the twenty-four hours, but the heat of the skin was always great, and the tongue always white; he had an acute pain always in his belly.

"The first step was to purge him briskly, but this made no change in the colour of his stools. The pupils now became large, accompanied with a complete strabysmus, and frequently flushings of the face: as his plaints from his head and belly were now becoming very intense, I ordered him gtt. vij. tinct. opii. to be repeated every three hours. I was greatly encouraged when I found him quite calm and placid under this medicine. When he had taken it five times, the dose was increased to eight drops every four hours. He complained a little of his belly during the night; but we had no more of those shocking yells from the shoots into his brain. The next morning I gave him his purge again, and repeated it every three hours till it had the full effect. After this the opiate was continued every four hours till his stools began to take their natural hue, which was in about three days; whether it was the opium which brought about these changes I cannot yet say; but it is worth the while of those who attend children's dispensaries to make themselves fully acquainted with its mode of action till more is known.

"I had scarcely got the last case recovered before I was called to a little girl of four years old, in Darlington; but in this child the disease made more rapid progress at the commencement: one of her first symptoms was great heat and pain in her forehead, with pain in her belly, and very quick pulse, along with dark stools, and her bowels very slowly acted upon. Jalap, scammony, and calomel, were now the order of the day; but these were attended with little relief, and no change of colour. The pupils were generally large when in health, and her hair light coloured; she was much inclined to keep her bed, and her pulse, from being one hundred and thirty, had now got to be a little more than sixty, with a palpable strabysmus, and the pupils much enlarged. I was resolved to delay no longer, but gave her tinct. opii. gtt. ix. and repeated this dose every three hours; except every now and then we rested upon our oars for the sake of giving the purgatives: but, from the time of the opiate being given, there was an evident relief from pain, and the parents of the child made this remark with great joyfulness. I have the satisfaction to state, that in the third day of giving the opiate her stools got more natural, and she had no more pain." (P. 37.)

Our author, in a communication with which he favoured us about a twelvemonth since, and which appeared in an abridged form in our second Number, p. 440, asserted that calomel and cold water are infallible specifics in cholera; and here he says the same thing over again.

"But as soon as we administered a grain of calomel every ten minutes, washing it down with a draught of cold spring water, we did not lose one patient, although several of them did not send for assistance till their hands and feet, and some of them their faces, were a deep black blue; we had twenty-three cases in August, and before the disease left the country we had thirty, not one of whom died; although there were spiteful and unprincipled people to be found who were ready to say that we had not the Asiatic cholera in Darlington. But their motive for saying so was very good to understand. Had two-thirds of them died, as was the case in many places, they would have been ready enough to allow it to have been cholera. But I have often remarked, that I have made more enemies by a successful practice than an adverse. Were there need, I could produce a host of evidence that many of our patients had dark blue extremities, with every other symptom of Asiatic cholera, and recovered. What do the sceptics say to this?" (P. 49.)

Now we, who are not sceptics as to Dr. Peacock's good intentions, but only as to his infallibility,—we, who are not "spiteful and unprincipled people," but, on the contrary, the very essence of honesty and goodhumour, will explain to our author why all the world does not treat cholera with calomel and cold water.

The fact is, that, were we to lend a ready ear to doctors' laudations of new remedies, we should find that we were theoretically possessed of at least six specifics for every known disease. Taught by a thousand disappointments, we listen to the physician's tale of marvels with the same feelings with which we hear a fond parent's praise of the cleverness of his child. It is in vain that he tells us that John is the sharpest fellow in London; we want to hear his evidence confirmed by colder judges. It is just the same in physic; we cannot possibly doubt a gentleman's word, but still we would wish to have the remedy tried by other physicians. But, when this has been done, how unsatisfactory is too often the result. If you want one instance out of ten thousand, just open Thomson's Dispensatory, at the article *Toxicodendri folia,* and read of their properties and uses. "The leaves are stimulant and narcotic. In the hands of Dr. Alderson, of Hull, who introduced them as a remedy, they proved successful in several cases of paralysis; but we believe their efficacy in this disease has not been confirmed by the observations of other physicians." (P. 531, 4th edition.) These illusory specifics, which fall to the ground one after another, as numerous and as evanescent as the leaves in autumn, make us sceptical even as to real ones, which come, "like angels' visits, few and far between." We are disposed to try their merits by severe tests, but truth does not dread them; just as, in a great town, every stranger is taken to be a rogue, but no honest man is offended that he is suspected.

Now, when a practice is so amazingly successful as Dr. Peacock's, we should expect that the most envious doctor in Darlington must cry "Euge!" or, at any rate, that the public, who have no great relish for medical squabbles, but who can tell if a patient died or not, would solicit our author's attendance at every cholera case within forty miles. Nay, more; we should expect that other doctors, not absolutely in Darlington, (for their motives, as our author says, "are very good to understand,") but pretty near it, would be compelled for very shame to save their patients; or that, if they too were pitiless, that the laity of Durham would take the calomel into their own hands, and refuse to die by rule.

As these natural events, however, do not seem to have happened, we are compelled to suppose that our author was in some degree misled by his partiality for the remedy he had introduced; and we take to this supposition, because we always think it more probable that one physician should be mistaken than a dozen.

We next come to a case of hydatids.

"A lady in the north, somewhat connected with the navy, had been sometime pestered with these equivocal beings, for which she had been in the habit of pouring in broadsides of the most drastic purgatives, which generally brought some away. They occupied the whole of the intestinal canal, but luckily had not found out any of the other cavities, but she could accurately distinguish where they were; they occupied the upper part of the gullet, and the whole of the stomach and intestines. As soon as I was honored with the command, I was resolved to '*board*' them, which I did very promptly with large doses of the Calais sand of the shops, stirred up in treacle, and mixed with the briskest purgatives in our possession. The enemy soon 'surrendered at discretion,' and in greater numbers than I had any previous conception of. The lady has had no return." (P. 50.)

Our author gives the following pills as a specific for headachs of all kinds:

"℞. Bac. Capsici, ℈ij.; Gum Arabic, gr. x.; Syr. Alb. q. s. ft. pil. xx. quar. sum iv. 2da quaq. hora dolore urgenti."

We see no objection to these pills in the ordinary atonic headach, so common in London; but of what benefit could they be when the headach proceeds from a loaded stomach, or a diseased brain?

Dr. Peacock has been very successful in treating lupus, but he is afraid that the doctors will pounce upon these cases too, and analyze, and criticise, and distinguish, till they have shown that a wolf is a lamb.

"But I presume all these cases, like the successful cases of cholera, will be disputed, because they were successful. However, if men of candour and industry, who have the care of infirmaries and dispensaries, will have the goodness to make a trial of the following remedy, I think they will be pleased with it. The part affected is smeared with it two or three times a day, either by the finger, or, if the diseased part is very sensible, by means of a camel's hair pencil.—℞. Axung. Porcin. ℥j.; Sulph. Prœcip. ʒss.; Hydrar. Sulph. rubr. gr. x. M. ft. ung.

"As a medicine, I find the following pill, taken three times a day, alleviate the suffering very much.—℞. Opii.; Pulv. Digitalis. an gr. ss.; Syr. q. s. M. ft. Pil. j.

"Should the pulse become intermittent after taking this pill a few days, then to take it only twice a day." (P. 71.)

This little work does honour to Dr. Peacock's talents as well as industry, and will be consulted as a repertory of remarkable cases and judicious practice.

